# Viral Metagenomics on Animals as a Tool for the Detection of Zoonoses Prior to Human Infection?

**DOI:** 10.3390/ijms150610377

**Published:** 2014-06-10

**Authors:** Sarah Temmam, Bernard Davoust, Jean-Michel Berenger, Didier Raoult, Christelle Desnues

**Affiliations:** Unité de Recherche sur les Maladies Infectieuses Tropicales Emergentes (URMITE) UM63, CNRS 7278, IRD 198, INSERM 1095, Aix-Marseille Université, 13005 Marseille, France; E-Mails: sarah.temmam@gmail.com (S.T.); bernard.davoust@gmail.com (B.D.); jean-michel.berenger@ap-hm.fr (J.-M.B.); didier.raoult@gmail.com (D.R.)

**Keywords:** viral metagenomics, hematophagous arthropods, zoonoses, wildlife, domestic animals

## Abstract

Many human viral infections have a zoonotic, *i.e.*, wild or domestic animal, origin. Several zoonotic viruses are transmitted to humans directly via contact with an animal or indirectly via exposure to the urine or feces of infected animals or the bite of a bloodsucking arthropod. If a virus is able to adapt and replicate in its new human host, human-to-human transmissions may occur, possibly resulting in an epidemic, such as the A/H1N1 flu pandemic in 2009. Thus, predicting emerging zoonotic infections is an important challenge for public health officials in the coming decades. The recent development of viral metagenomics, *i.e.*, the characterization of the complete viral diversity isolated from an organism or an environment using high-throughput sequencing technologies, is promising for the surveillance of such diseases and can be accomplished by analyzing the viromes of selected animals and arthropods that are closely in contact with humans. In this review, we summarize our current knowledge of viral diversity within such animals (in particular blood-feeding arthropods, wildlife and domestic animals) using metagenomics and present its possible future application for the surveillance of zoonotic and arboviral diseases.

## 1. Introduction

Human microbiologic infections, known as zoonoses, are acquired directly from animals or via arthropods bites and are an increasing public health problem. More than two thirds of emerging human pathogens are of zoonotic origin, and of these, more than 70% originate from wildlife [1,2]. In novel environments, viruses, particularly RNA viruses, can easily cross the species barrier by mutations, recombinations or reassortments of their genetic material, resulting in the capacity to infect novel hosts. Because of their adaptive abilities, RNA viruses represent more than 70% of the viruses that infect humans [3]. When socio-economic and ecologic changes affect their environment, humans may encounter increased contact with emerging viruses that originate in wild or domestic animals.

**Figure 1:**
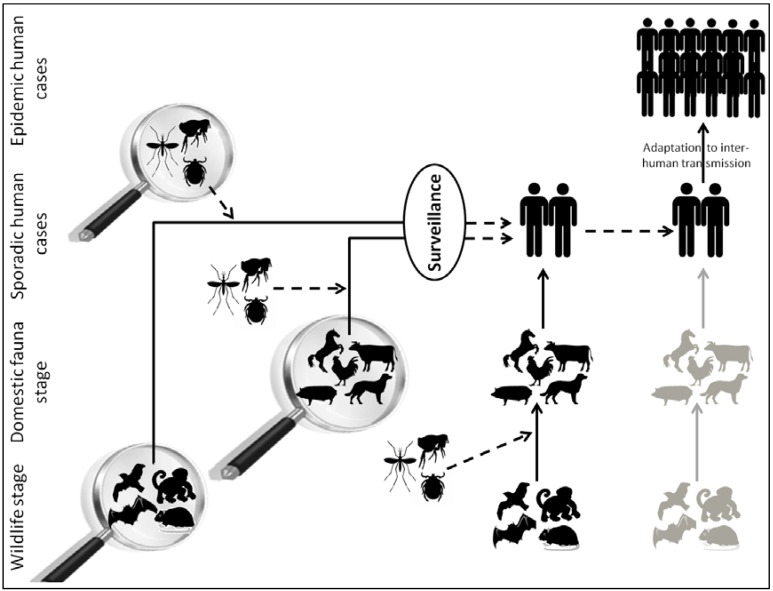
The origins of zoonotic human infections.

Wolfe *et al.* in 2007 [4] and Karesh *et al.* in 2012 [5] described different stages in the switch from an animal-specific infectious agent into a human-specific pathogen. The key stage is the transition of a strictly animal-specific infectious agent (originating from wildlife or domestic animals) to exposed human populations, resulting in sporadic human infections (Figure 1). If the pathogen is able to adapt to its human host and acquire the means to accomplish an inter-human transmission, horizontal human-to-human transmission occurs and maintains the viral cycle. Sometimes, an intermediate host, such as a domestic animal, is the link between sylvatic viral circulation and human viral circulation. For example, some human infections originating from bats, such as Nipah, Hendra, SARS and Ebola viral infections, may involve intermediate amplification in hosts such as pigs, horses, civets and primates, respectively [6] (Figure 1). Genetic, biologic, social, political or economic factors may explain a switch in viral host targets. For example, climate changes may influence the geographical repartition of vector arthropods, leading to new areas of the distribution of infectious diseases, like *Aedes albopictus* and Chikungunya infections in the Mediterranean [7]. Morens *et al.* [8] listed different key factors that may contribute to the emergence or re-emergence of infectious diseases, such as microbial adaptation to a new environment, biodiversity loss, ecosystem changes that lead to more frequent contact between wildlife and domestic animals or human populations, human demographics and behavior, economic development and land use, international travel and commerce, *etc.* [9,10]. These patterns of transmission allow identifying different animals to follow in order to monitor the appearance of new or re-emerging infectious agents before its first detection in the human populations. Therefore, hematophagous arthropods, wildlife and domestic animals may serve as targets for zoonotic and arboviral disease surveillance, particularly because sampling procedures and long-term follow-up studies are more easily performed in these hosts than in humans.

Historically, classic viral detection techniques were based on the intracerebral inoculation of suckling mice or viral isolation in culture and the subsequent observation of cytopathic effects on cell lines. Later, immunologic methods, e.g., seroneutralization or hemaglutination, were used to detect viral antigens in various complex samples. These techniques were based on the isolation of viral agents. With the progresses of molecular biology, polymerase chain reaction (PCR)-based methods became the main techniques for virus discovery and allowed the detection of uncultivable viruses [11], but these techniques required prior knowledge of closely related viral genomes. Next-Generation Sequencing (NGS) techniques make it possible to sequence all viral genomes in a given sample without previous knowledge about their nature. These techniques, known as viral metagenomics, have allowed the discovery of completely new viral species. Because of their low cost, the use of NGS techniques is exponentially increasing.

The transmission of infections between humans occurs after a pathogen from a wild or domestic animal contacts with exposed human populations. The human exposures may or may not be mediated by the bite of bloodsucking arthropods. Surveillance programs may target wildlife, domestic animals or arthropods for emerging viruses before their adaptation to human hosts.

## 2. Viral Metagenomics: A Powerful Technique to Inventory the Viral Diversity among Complex Environments

Viruses can be identified by a wide range of techniques, which are mainly based on comparisons with known viruses. Historic methods include electron microscopy, cell culture, inoculation in suckling mice and serology, but these methods have limitations. For example, many viruses cannot be cultivated, excluding the use of cell line isolation and serologic techniques, and can only be characterized by molecular methods. In 2011, Bexfield summarized the different molecular techniques that identify new viruses such as microarray, subtractive hybridization-based and PCR-based methods [12]. Although these techniques have allowed the discovery of many viruses, the prior knowledge of similar viruses is required. Recent advances in sequence-independent PCR-based methods have overcome this limitation, and Sequence-Independent Single Primer Amplification (SISPA), Degenerate Oligonucleotide Primed PCR (DOP-PCR), random PCR and Rolling Circle Amplification (RCA) methods have emerged [12]. The end result of most of these PCR methods is amplified DNA that requires definitive identification by sequencing.

Novel DNA sequencing techniques, known as “Next-Generation Sequencing” (NGS) techniques, are new tools providing high-throughput sequence data with many possible applications in research and diagnostic settings [13]. With the development of different NGS platforms, it is now possible to sequence all viral genomes in a given sample without previous knowledge about their nature with the use of sequence-independent amplification followed by high-throughput sequencing. This combination of techniques, known as viral metagenomics, allows the discovery of completely new viral species within a complex sample and, due to decreasing costs, are nowadays exponentially increasing.

NGS techniques are able to generate a huge number of sequences, ranging from thousands to millions of reads, in only one reaction. In order to fully benefit from this depth of sequencing to identify infectious agents present in a given environment, host DNA/RNA should previously be removed from samples. Preliminary treatments are therefore required prior to nucleic acid amplification and sequencing, mainly based on nucleases treatments and/or viral purification by ultracentrifugation on sucrose, cesium chloride or glycerol gradients. These strategies are known as “Particle-Associated nucleic acid amplification” [14], *i.e.*, they try to isolate intact (*i.e.*, infectious) viral particles from their environment, protected from the action of nucleases. Subsequent low amount of nucleic acids have required the use of Sequence-Independent Amplifications (SIA) such as SISPA, DOP-PCR, random PCR, RCA [12]. Although these techniques allow generating enough nucleic acid material for sequencing, their main disadvantage remains that they distort quantitative analyzes by introducing bias of amplification in viral diversity studies. As a consequence, quantitative analyses of the composition of resulting viromes may not reflect the reality.

In diagnostic virology, in either human or veterinary medicine, viral metagenomics has allowed the discovery of causative viral agents of disease conditions [13,14,15,16,17]. Virome analyses have also been conducted to describe the baseline viral diversity in healthy human conditions [18], as a prior knowledge before studying the viral flora of pathologic conditions.

In the same way, the use of viral metagenomics as a tool for arboviral and zoonotic disease surveillance requires prior knowledge of the viral diversity associated to hematophagous arthropods and animals in close contact with humans. This review thus summarizes our current knowledge of the diversity of viral communities associated with several arthropods, wildlife and domestic animals and present its potential applications for the surveillance of zoonotic and arboviral diseases.

## 3. Blood-Feeding Arthropods

Several species of arthropods require a blood meal for their survival, either for their gonotrophic cycle or for feeding. Because humans and arthropods may share a common habitat, arthropods may feed on humans. When an arthropod feeds on a vertebrate host, it injects saliva into the host’s blood and, if the micro-organism was previously able to replicate and to migrate into the salivary glands of the arthropod, the micro-organism is concomitantly injected to the vertebrate host’s blood when the arthropod feeds. Such arthropods are called vectors. Medical entomology has therefore studied vector-host relationships for the last century. Several human pathogenic micro-organisms, including a large number of viral families, are transmitted via the bite of a bloodsucking arthropod. Reservoirs may be the hematophagous arthropod itself if viruses are maintained by trans-ovarial and trans-stadial transmission to the progeny, or in several cases, the reservoirs may be of wildlife or domestic animal origin, with arthropods providing the transmission of the virus between the two vertebrate hosts. Table 1a summarizes the principal hematophagous arthropods of medical importance and associated arboviruses (for arthropod-borne viruses) detected by classic methodologies.

Recently, the use of metagenomics has allowed the detection of a large number of known and unknown insect-specific or zoonotic viruses associated with arthropods [19,20]. Broad surveys of viral diversity in arthropods are lacking, although metagenomics is a powerful technique for these studies and may be a promising tool for arbovirus surveillance. Only a few studies, focused on mosquitoes, are available. The major viruses that have been detected may be summarized in 4 categories (Table 1b): insect-specific viruses, plant viruses reflecting the nectar meal of mosquitoes, bacteria-infecting viruses (bacteriophages) and animal viruses possibly reflecting the arthropod blood meal of the vertebrate host.

### 3.1. Zoonotic Viruses

Ng and collaborators, in 2011, were the first to conduct a wide survey of viral diversity within mosquitoes using metagenomics [21]. Viral reads represented only 1% to 2% of total reads obtained after 454 pyrosequencing, and animal viruses represented not more than 10% of viral reads. Several mosquito species do not have strict host-specific trophic preferences, such as *Culex erythrothorax*, which was studied by Ng, *et al*. [21]*.* As a consequence, animal viruses detected in mosquitoes possibly reflect the virome of the large variety of vertebrate hosts they fed on (e.g., humans, primates, birds). For example, *Papillomaviridae* detected in mosquitoes have a human origin, while *Circoviridae* have mainly a bird origin [21]. No potential zoonotic viruses were documented in this study.

In 2014, Coffey and collaborators conducted a metagenomic analysis of Australian mosquitoes [22]. They were able to detect many animal viruses belonging to the *Flaviviridae* (Edge Hill virus) and *Reoviridae* (Wallal virus) families, but several viruses described in the study are known to infect marsupials. The authors also detected Ross River virus, an *Alphavirus* belonging to the *Togaviridae* family transmitted by mosquitoes, which is able to infect humans and cause influenza-like illness and/or polyarthritis, mainly in Australia. Coffey *et al.* also reported a novel virus, a dipteran-mammal-associated rhabdovirus called dimarhabdovirus. Viruses belonging to this supergroup are known to replicate both in hematophagous dipterans and vertebrate hosts [23], but no evidence of human infections are reported. Sequences belonging to the *Orthobunyavirus* genus (*Bunyaviridae* family) were also detected [22]. *Bunyaviridae* are single-stranded negative-strand segmented RNA viruses infecting a wide variety of vertebrate and invertebrate hosts. The family comprises 5 genera, and the *Orthobunyavirus* genus is divided into 4 major groups (Bunyamwera, Wyeomyia, Simbu and California encephalitis). Phylogenetic analyses performed on L and M segments of the newly described orthobunyaviruses did not place them into a specific group, nor provided information regarding a vertebrate host origin. However, their identification in anthropophilic mosquitoes coupled with their ability to replicate in mammalian cells may presuppose a human or other mammalian origin.

Although most of the zoonotic viruses transmitted by arthropods are RNA viruses, the RNA virome of arthropods, especially mosquitoes, is poorly described and is a future challenge for arbovirus surveillance. Indeed, although studying viromes of engorged arthropods does not reflect the viral diversity intrinsically linked to arthropods, it provides a good picture of viruses circulating in a given human or animal population. The “One Health” concept (see below) recognizes that human health is linked to animal health and the environment in which they co-evolve. As a consequence and due to the ease with which they can be studied, arthropods may be used as targets for arboviral infections surveillance programs focusing either on human and veterinary health.

### 3.2. Insect-Specific Viruses

By sequencing the total DNA, Ng *et al.* [21] detected, 60% to 80% insect-specific viral reads, including a majority of densoviruses, as was also reported by Hall-Mendelin *et al.* [24]. Ma and collaborators [25] were also able to detect densoviruses by sequencing the small RNA molecules produced by the arthropod in response to viral infections. This process involved RNA interference or RNA silencing, resulting in the sequence-specific degradation of viral genomes.

Densoviruses (DNV) belong to the family *Parvoviridae*, sub-family *Densovirinae*. They are small, non-enveloped icosahedral ssDNA viruses, which infect the majority of arthropods (e.g., insect orders *Diptera*, *Hemiptera*, and *Lepidoptera*). DNV seem to be highly host-specific, and because they infect most tissues of their hosts, they are responsible for the death of their arthropod host. They apparently have larvicidal activity, and a few DNV-resistant adults may emerge, resulting in a population that is able to vertically transmit the infection to their descendants. In contrast, it seems that DNV infection of late-stage larvae or young adults results in the establishment of a persistent and vertically transmitted viremia. Thus, these viruses are a promising tool for vector control [26,27].

Other insect-specific viruses were detected by Cook *et al.* [28] using the same small RNA-based metagenomic technique, including *Chronic bee paralysis virus* and viruses belonging to the *Birnaviridae* family (genus *Entomobirnavirus*). The authors also detected sequences related to an insect-specific *Flaviviridae*, but it possibly constituted sequences integrated into the genome of the mosquito (Table 1b).

Table 1(**a**) Non-exhaustive list of major viruses detected in blood-feeding arthropods by various serological or molecular techniques; (**b**) Examples of viruses detected in mosquitoes by metagenomic studies.

**Table:** (**a**)

Kingdom	Class		Order		Family		Type Arthropod		Example of Viral Families		Ref.	
*Arthropoda*	*Arachnida*		*Ixodida*		*Ixodidae*		Hard tick		*Flaviviridae* (TBEV, OHFV) *Bunyaviridae* (CCHFV) *Reoviridae* (CTFV)		[29,30]	
					*Argasidae*		Soft tick		*Flaviviridae* (AHFV, SREV, WNV), *Bunyaviridae* (SOLV)		[31,32,33,34]	
	*Insecta*		*Anoploura*		*Pediculidae*		Louse		not documented			
			*Siphonaptera*		*Pulicidae*		Flea		*Flaviviridae* (TBEV)		[35]	
			*Hemiptera*		*Cimicidae*		Bed bug		*Bunyaviridae* (KKV) *Hepadnaviridae* (HBV)		[36,37,38,39]	
					*Reduviidae*		Triatoma		not documented			
*Arthropoda*	*Insecta*		*Diptera*		*Simuliidae*		Black fly		not documented			
					*Tabanidae*		Horse fly		*Bunyaviridae* (LACV, JCV)		[40,41]	
					*Psychodidae*		Sand fly		*Bunyaviridae *(SFNV, TOSV) *Flaviviridae*, *Rhabdoviridae*		[31,42,43,44,45,46]	
					*Muscidae*		Tsetse fly		not documented			
					*Culicidae*		Mosquito		*Flaviviridae *(WNV, YFV, DENV), *Togaviridae* (CHIKV, ONNV), *Bunyaviridae* (RVFV, NRIV)		[47,48,49,50]	
					*Ceratopogonidae*		Biting midge		*Bunyaviridae* (OROV, RVFV, CCHFV)		[51,52,53]	

Abbreviations: Tick-Borne Encephalitis virus (TBEV), Omsk Hemorrhagic Fever virus (OHFV), Colorado Tick Fever virus (CTFV), Alkhurma Hemorrhagic Fever virus (AHFV), Saumarez Reef virus (SREV), West Nile virus (WNV), Soldado virus (SOLV), Kaeng Khoi virus (KKV), Hepatitis B virus (HBV), Lacrosse virus (LACV), Jamestown Canyon virus (JCV), Sandfly Fever Naples virus (SFNV), Toscana virus (TOSV), Yellow Fever virus (YFV), Dengue virus (DENV), Chikungunya virus (CHIKV), O’nyong-nyong virus (ONNV), Rift Valley Fever virus (RVFV), Ngari virus (NRIV), Oropouche virus (OROV), Crimee-Congo Hemorrhagic Fever virus (CCHFV).

**Table:** (**b**)

Arthropod Species	Type Study	Viral Reads Taxonomic Assignation				Ref.
		Animal Viruses	Insect-Specific Viruses	Plant Viruses	Phages	
Mixed-species female mosquitoes	DNA virome	*Anelloviridae*, *Circoviridae*, *Herpesviridae*, *Poxviridae*, *Papillomaviridae*	*Parvoviridae* (*Densovirinae*), *Poxviridae* (*Entomopoxvirinae*), *Iridoviridae*	*Geminiviridae*, *Nanoviridae*	*Myoviridae*, *Podoviridae*, *Siphoviridae*	[21]
*Culex pipiens molestus*	Small RNA virome	not documented	*Parvoviridae* (*Densovirinae*)	not documented	not documented	[25]
*Aedes* sp.	DNA/RNA virome	not documented	*Parvoviridae* (*Densovirinae*)	not documented	*Inoviridae*	[24]
*Anopheles* sp., *Ochlerotatus* sp.	Small RNA virome	*Reoviridae* (*Orbivirus*) *	*Chronic bee paralysis virus*, *Birnaviridae* (*Entomobirnavirus*), *Flaviviridae* *, *Bunyaviridae* ** (*Phlebovirus*)	*Narnaviridae*, *Partitiviridae* *	not documented	[28]
*Anopheles* sp., *Culex* sp., *Aedes* sp.	DNA/RNA virome	*Rhabdoviridae*, *Bunyaviridae*, *Flaviviridae*, *Reoviridae*, *Togaviridae*	not documented	not documented	not documented	[22]

* Possibly integrated viral sequences into the genome of the arthropod; ** Classification hypothesized by the authors.

## 4. Wildlife

In 2004, Bengis *et al.* described two different patterns of transmission of infectious diseases from wildlife to humans [54]. The first pattern is one in which a viral disease of wildlife origin is transmitted rarely to humans, but once viral adaptation to the human host occurs, horizontal human-to-human transmission maintains the viral cycle. A major example of this pattern of transmission is the adaptation of HIV from SIV (Simian Immunodeficiency Virus) [55]. The second pattern involves many animal-to-human transmission events, possibly mediated by arthropods, for which animals are reservoirs and horizontal human-to-human transmissions are rare (Figure 1). A good example is West Nile virus infection, for which the usual viral cycle involves wild birds, mosquitoes, rarely horses and humans, who are accidental hosts [56]. Table 2a summarizes the principal zoonotic viruses associated with wildlife that are able to infect humans.

### 4.1. Bats

Frugivorous, insectivorous or hematophagous bats worldwide have been studied for their role as reservoirs of infectious agents. Many viruses isolated from bats are able to cross the species barrier and infect humans, regularly causing severe diseases in humans (e.g., SARS, Ebola hemorrhagic fever, Nipah, rabies) (Table 2a). Most metagenomic studies targeting wildlife have been conducted on bats (Table 2b), as Calisher and collaborators reviewed in 2006 [57], Wong and collaborators in 2007 [6], Smith and Wang in 2013 [58] or Luis *et al.* in 2013 [59]. Because “bat science” is a large and well-studied area in infectious diseases, this review will not focus more on this topic.

### 4.2. Rodents

Because of their close contact with humans, rodents are known reservoirs of pathogens, including many viral families (Table 2a). The major source of human contact with rodent pathogens is the exposure to the urine or feces of infected animals via the environment.

To describe the viral diversity of feces of wild rodents living in contact with humans, Phan and collaborators conducted a metagenomic analysis of 105 fecal specimens from mice, voles and rats [60]. They reported the presence of insect (e.g., *Densovirinae*, *Iridoviridae*) and plant viral sequences (e.g., *Nanoviridae*, *Geminiviridae*) reflecting the diet of rodents (Table 2b). They also detected several mammalian viruses, including the first known mouse sapelovirus and astrovirus, a species-specific mouse papillomavirus and novel picornaviruses possibly forming new genera within the family. Based on phylogenetic and distance-based data, a close relative of the Aichi virus was discovered in the murine stool samples [60]. Aichi virus is a virus belonging to the *Picornaviridae* family that has been identified in human diarrheas but for which the pathogenicity has not clearly been demonstrated. Although the murine Aichi virus shared more than 80% identity with the human Aichi virus, further studies need to be conducted to determine if this new virus is able to infect humans and, because it is excreted in rodents’ feces, may represent a potential threat to human health.

Phan *et al.* also noted the presence of plant viruses, such as *Virgaviridae*, in the virome of the rodents’ feces [60]. The authors concluded that these viruses reflect the diet of rodents, and usually plant viruses are considered incapable of infecting humans. However, a few studies reported the presence of plant viral RNA in the human body, including the respiratory system via the use of cigarettes [61] and the gut via the consumption of contaminated food [62] though there is no evidence of a role in human pathologies.

Whether the animal viruses detected in the studies conducted on target animals have the capacity to infect humans is, to our knowledge, unknown, and this capacity needs to be further characterized before developing a metagenomic-target-based tool that is useful for the surveillance of emerging zoonoses.

Table 2(**a**) Non-exhaustive list of major zoonotic viruses detected in wildlife; (**b**) Examples of viruses detected in bats and rodents by metagenomic studies.

**Table:** (**a**)

Wildlife	Zoonosis	Virus	Vector-Based Transmission	Domestic Animal Intermediate Host	Ref.
Bat	Nipah/Hendra	*Paramyxoviridae*, *Henipavirus*	No	Pig/horse	[63]
	Ebola hemorrhagic fever	*Filoviridae*, *Ebolavirus*, EBOV	No	No	[64]
	Severe acute respiratory syndrome (SARS)	*Coronaviridae*, *Betacoronavirus*, SARS-CoV	No	Civet, cat	[65]
	Rabies	*Rhabdoviridae*, *Lyssavirus*, RABV	No	Dog	[66]
Rodent	Lymphocytic choriomeningitis	*Arenaviridae*, *Arenavirus*, LCMV	No	No	[67]
	Lassa hemorrhagic fever	*Arenaviridae*, *Arenavirus*, LASV	No	No	[67]
	Pulmonary syndrome and hemorrhagic syndrome	*Bunyaviridae*, *Hantavirus*	No	No	[68,69]
Bird	Japanese encephalitis	*Flaviviridae*,* Flavivirus*, JEV	Yes (mosquitoes)	Swine	[70]
	West Nile	*Flaviviridae*, *Flavivirus*, WNV	Yes (mosquitoes)	Horse	[56]
	Avian influenza	*Orthomyxoviridae*, *Influenzavirus*, A/H5N1, A/H1N1	No	Poultry, swine	[71,72,73]
Primate	Marburg hemorrhagic fever	*Filoviridae*, *Marburgvirus*, MARV	No	No	[74,75]
	Acquired immunodeficiency syndrome (AIDS)	*Retroviridae*, *Lentivirus*, HIV	No	No	[55]

Abbreviations: Ebola virus (EBOV), SARS-Coronavirus (SARS-CoV), Rabies virus (RABV), Lymphocytic ChorioMeningitis virus (LCMV), Lassa virus (LASV), Japanese Encephalitis virus (JEV), West Nile virus (WNV), Marburg virus (MARV), Human Immunodeficiency Virus (HIV).

**Table:** (**b**)

Wild Animals	Type Study	Example of the Taxonomic Assignation of Viral Reads				Ref.
		Animal Viruses	Plant/Fungal Viruses	Phages	Insect-Specific Viruses	
Bats	DNA/RNA virome (feces)	*Parvoviridae*, *Circoviridae*, *Picornaviridae*, *Adenoviridae*, *Poxviridae*, *Astroviridae*, *Coronaviridae*	*Luteoviridae*, *Secoviridae*, *Tymoviridae*, *Partitiviridae*	*Microviridae*, *Siphoviridae*	*Dicistroviridae*, *Iflaviridae*, *Tetraviridae*, *Nodaviridae*, *Parvoviridae* (*Densovirinae*)	[76]
	DNA/RNA virome (feces, urine, throat swabs, tissue)	*Coronaviridae*, *Herpesviridae*	*Tymoviridae*	*Podoviridae*	*Iflaviridae*, *Dicistroviridae*	[77]
	DNA/RNA virome (feces, urine, tissue, serum, throat swabs)	*Retroviridae*, *Flaviviridae*, *Caliciviridae*, *Togaviridae*, *Paramyxoviridae*, *Adenoviridae*, *Papillomaviridae*, *Parvoviridae*, *Herpesviridae*, *Hepadnaviridae*	not documented	not documented	not documented	[78,79]
	DNA/RNA virome (feces)	*Papillomaviridae*, *Circoviridae*, *Anelloviridae*	not documented	unclassified	*Parvoviridae* (*Densovirinae*)	[80]
	DNA/RNA virome (feces, throat swabs)	*Adenoviridae*, *Herpesviridae*, *Papillomaviridae*, *Retroviridae*, *Circoviridae*, *Rhabdoviridae*, *Astroviridae*, *Flaviviridae*, *Coronaviridae*, *Picornaviridae*, *Parvoviridae*	*Chrysoviridae*, *Hypoviridae*, *Partitiviridae*, *Totiviridae*	*Inoviridae*, *Microviridae*	*Baculoviridae*, *Iflaviridae*, *Dicistroviridae*, *Tetraviridae*, *Parvoviridae* (*Densovirinae*)	[81]
	DNA/RNA virome (tissue)	*Herpesviridae*, *Papillomaviridae*, *Polyomaviridae*, *Hepadnaviridae*, *Circoviridae*, *Poxviridae*, *Retroviridae*, *Astroviridae*	*Phycodnaviridae*, *Bromoviridae*	*Myoviridae*, *Podoviridae*, *Siphoviridae*	*Baculoviridae*, *Polydnaviridae*, *Parvoviridae* (*Densovirinae*), *Iflaviridae*	[82]
	DNA/RNA virome (urine, throat swabs)	*Herpesviridae*, *Papillomaviridae*, *Adenoviridae*, *Poxviridae*,*Polyomaviridae*, *Retroviridae*, *Parvoviridae*, *Picornaviridae*	not documented	not documented	*Parvoviridae* (*Densovirinae*)	[83]
Rodents	DNA/RNA virome (feces)	*Circoviridae*, *Picobirnaviridae*, *Picornaviridae*, *Astroviridae*, *Parvoviridae*, *Papillomaviridae*, *Adenoviridae*, *Coronaviridae*	*Nanoviridae*, *Geminiviridae*, *Phycodnaviridae*, *Secoviridae*, *Partitiviridae*, *Tymoviridae*, *Alphaflexiviridae*, *Tombusviridae*	unclassified	*Parvoviridae* (*Densovirinae*), *Iridoviridae*, *Polydnaviridae*, *Dicistroviridae*, *Bromoviridae*, *Virgaviridae*	[60]

## 5. Domestic Animals

Several human infections have their origin in domestic animals. For example, the reservoir of genotype-3 Hepatitis E virus is pigs, but humans may be infected by the consumption of undercooked contaminated meat [84]. Some of these zoonotic viruses may be vector-transmitted to humans, such as the Rift Valley fever virus (Table 3a).

In veterinary medicine, metagenomic studies were conducted chiefly to determine the causal agent of pathologies with unknown etiology [16,17,85,86]. Few studies were conducted to describe the viral flora within domestic animals without a pathologic context. The viral diversity of domestic animals is summarized in Table 3b, which lists the major viruses discovered by veterinary medicine through metagenomics. Analyzing viral circulation within the domestic animals by metagenomics is a promising tool not only for veterinary medicine but also for the surveillance of zoonotic viruses possibly transmissible to humans for whom domestic animals act as reservoirs or intermediate hosts between wildlife and humans. Following the circulation of known and potential emerging viral agents in domestic animals appears to be an important surveillance goal.

### 5.1. Swine Breeding

Shan *et al.* reported in 2011 the metagenomic analysis of feces from healthy and diarrheic piglets grown in a high-density farm [87]. Viral reads represented 64% to 68% of total reads obtained after 454 sequencing, and RNA viruses accounted for more than 98% of viral reads. Beyond the RNA viral families detected, *i.e.*, *Picornaviridae*, *Astroviridae*, *Caliciviridae*, and *Coronaviridae* families, no zoonotic viruses that could infect humans were detected (Table 3b). In fact, the authors noted the presence of kobuviruses, astroviruses, enteroviruses, sapoviruses, sapeloviruses, coronaviruses, bocaviruses and teschoviruses either in healthy or diarrheic piglets, with only variations in the number of reads between healthy and diarrheic piglets. They concluded that such co-infections, even in healthy animals, may promote recombinations or reassortments between viruses, resulting in the emergence of new viruses, possibly infecting humans.

In 2012, Masembe and collaborators conducted a metagenomic analysis of domestic pig sera as part of a routine general surveillance program for African swine fever [88]. They were able to detect not only strictly swine-specific viruses (such as African swine fever viruses or swine Torque Teno viruses) but also zoonotic arboviruses (Table 3b). In fact, they reported the presence of Ndumu virus, an *Alphavirus* transmitted by mosquitoes, which may infect cattle [89] and humans [90] for whom no symptoms are yet known. Thus, the study of Masembe and collaborators emphasizes the usefulness of using metagenomics on domestic animals as a tool for the surveillance of human-infecting arboviruses.

### 5.2. Bushmeat and Wild Boars

Illegal bushmeat traffic is a problem for biodiversity conservation and is also a potential threat to human health when contaminated tissues are consumed [91]. Even legal bushmeat is a potential infectious hazard.

Bushpigs are hunted African wild boars which, because of their increasingly exploited habitat, have increased contact with domestically bred pigs and may infect domestic animals living in close contact with humans. Bushpig meat is also consumed in some African countries. In this context, Blomström and collaborators conducted a metagenomic analysis of bushpig sera collected in Uganda [92]. They detected the presence of sequences related to suid-specific viruses, such as new variants of Porcine Parvovirus 4 and Torque Teno sus viruses, and the presence of a transcriptionally active Porcine Endogenous retrovirus. No zoonotic viruses, possibly infecting humans, were reported.

Reuter *et al.* conducted a similar study on wild boar feces collected in Hungary [93]. They noted the presence of viral reads matching the porcine Kobuvirus, a close relative of the human Aichi virus. Aichi virus and Kobuvirus are both viruses belonging to the *Picornaviridae* family detected in human and swine diarrheas respectively but for which the pathogenicity is not yet clearly demonstrated. Further studies should be conducted to determine whether porcine or wild boar kobuviruses are highly host-specific or if these viruses are able to infect humans.

Table 3(**a**) Non-exhaustive list of major viruses detected in blood-feeding arthropods by various serological or molecular techniques; (**b**) Examples of viruses detected in mosquitoes by metagenomic studies.

**Table:** (**a**)

Domestic Animal	Zoonosis	Virus	Vector-Based Transmission	Ref.
Cats, dogs	Rabies	*Rhabdoviridae*, *Lyssavirus*, RABV	No	[94,95]
Cattle, sheep, goats	Rift Valley fever	*Bunyaviridae*, *Phlebovirus*, RVFV	Yes (mosquitoes)	[96,97]
	Vaccinia	*Poxviridae*, *Orthopoxvirus*, VACV	No	[98]
Pigs	Hepatitis E	*Hepeviridae*, *Hepevirus*, HEV	No	[99]
	Japanese encephalitis	*Flaviviridae*, *Flavivirus*, JEV	Yes (mosquitoes)	[70]
Horses	West Nile	*Flaviviridae*, *Flavivirus*, WNV	Yes (mosquitoes)	[56]
	Hendra	*Paramyxoviridae*, *Henipavirus*, HeV	No	[63]
Poultry	Avian flu	*Orthomyxoviridae*, *Influenzavirus*, A/H5N1	No	[72,73]

Abbreviations: Rabies virus (RABV), Rift Valley Fever virus (RVFV), Vaccinia virus (VACV), Hepatitis E virus (HEV), Japanese Encephalitis virus (JEV), West Nile virus (WNV), Hendra virus (HeV).

**Table:** (**b**)

Animal Species	Type Studies	Viral Reads Taxonomic Assignation		Ref.
		Animal Viruses *stricto sensu*	Zoonotic Viruses	
Pigs	DNA/RNA virome (serum)	*Asfarviridae*, *Anelloviridae*, *Retroviridae*	*Togaviridae* (*Alphavirus*)	[88]
	DNA/RNA virome (stool)	*Picornaviridae*, *Astroviridae*, *Caliciviridae*, *Coronaviridae*, *Circoviridae*, *Parvoviridae*	not documented	[87]
Bushpigs	DNA/RNA virome (serum)	*Parvoviridae*, *Circoviridae*, *Retroviridae*	not documented	[92]
Wild boars	DNA/RNA virome (feces)	*Picornaviridae*, *Astroviridae*	not documented	[93]

No bacteriophages or plant viruses have been reported yet.

## 6. Future Perspectives in Metagenomic-Based Surveillance Programs

Viruses are the most abundant biological entities in the environment, including in the human body [18]. Viruses make up over two-thirds of all new human pathogens, a highly significant over-representation given that most current human pathogen species are bacteria, fungi or helminthes [100]. There are 219 viral species (belonging to 23 families) that are known to infect humans, among which more than two-thirds are of zoonotic origin [3].

Rudolf Virchow (1821–1902), a German physician and pathologist said “between animal and human medicine there are no dividing line, nor should there be”. Although more than 60% of viruses that infect humans are of zoonotic origin, human and veterinary medicine has each evolved separately until recently. Only recently physicians and researchers working on human infectious diseases have become aware that human interactions with the ecosystem may affect human health. As a consequence, an interdisciplinary approach to health has begun that includes physicians, researchers, veterinarians, epidemiologists, and ecologists. This recent strategy, known as the “One World, One Health” concept [101] seeks to increase communication, collaboration, and cooperation across a wide variety of disciplines, such as human and veterinary medicine, public health, microbiology, and ecology, to attain optimal health for people, animals and the environment in which they evolve.

In this context, zoonotic-borne and arbovirus-borne disease surveillance programs have recently integrated entomology and veterinary medicine. To prevent emerging infectious diseases in humans, surveillance programs now focus on the early detection of new or re-emerging infectious agents in hematophagous arthropods and wild or domestic animals, before viral adaptation to human hosts (Figure 1). Viral metagenomics are well-adapted tools for these surveillance programs because they allow the detection of all viral genomes in a given sample without previous knowledge of their nature.

Because they are easy to sample, arthropods may be used as targets for emerging arbovirus-borne disease surveillance. Recent metagenomic analyses focused on mosquito arthropods have demonstrated the richness of the mosquito virome, including viruses that reflect the nectar or blood meals [19,20] (Table 1b). Because arboviruses are transmitted to vertebrate hosts via the saliva of arthropods, a simple way to determine if emerging viral pathogens may be transmitted to humans is to selectively analyze the virome of the salivary glands of the arthropod, even though dissection is difficult for extremely small arthropods. However, metagenomic studies targeting the entire body of the bloodsucking arthropod not only allow for the description of the viral flora within the arthropod, which highlight the emerging infectious agents or insect-specific viruses as tools for vector population control, but they also allow for the study of interactions between viral and bacterial communities that may result in viral interference (e.g., Wolbachia endosymbiont and Dengue virus interactions [102,103]) This information can lead to the development of new antiviral strategies. Because detecting viruses from the entire arthropod does not conclusively mean there is vector-based transmission of viruses to vertebrates, these studies would therefore require determining whether the virus is able to multiply in the arthropod and to migrate into the salivary glands.

Wild fauna may also be appropriate target animals for emerging zoonoses surveillance. Because of the many restrictions on studying endangered wild animals (such as bats), non-invasive sampling procedures may be used such as collecting urine or feces. Moreover, humans are more frequently in contact with feces or urine of wild animals in their shared environment, rather than with tissues or blood, with the exception of the consumption of bushmeat. As a consequence, most metagenomic studies conducted on wildlife have involved the feces or urine of wild animals (Table 2b) [58,60]. As for arthropods, these studies revealed how diverse and species-specific is the virome, and how unknown viruses have yet to be discovered.

Recent studies searching for the reservoir of Middle-East Respiratory Syndrome-Coronavirus (MERS-CoV) have shown the potential role of camels in the transmission of MERS-CoV to humans [104,105]. Camels are not the usual targets of zoonotic surveillance programs, but these recent examples highlight the interest of focusing future viral metagenomic studies on other animal species interacting with humans if one considers their ability to transmit human infectious agents by crossing the species barriers between animals and humans.

Metagenomics is thus a promising tool for the detection of new viral species that could potentially be a threat for human health. However, it yet suffers several pitfalls when considering new/highly divergent viral genomes. Indeed, the taxonomic assignation of reads generated by NGS techniques is only based on the comparison of sequences or patterns with previously described sequences present in databases. As a consequence, completely new or highly divergent viral sequences might be difficult to identify and subsequently there is a high risk to miss the detection of important viral pathogens. This problem remains the major challenge of metagenomic studies. Future progress in metagenomics should improve *in silico* analyses to overcome or attenuate this problem and would therefore permit to use metagenomics tools for the surveillance of emerging viruses.

Finally, detecting viruses, and especially viral genomes, within a given animal does not provide evidence of the transmissibility of the virus to humans. Determining the viral ability to cross the species barrier and to infect humans is a necessary part of studying viral metagenomics. In 1890, Koch’s postulates described 4 criteria to determine the etiology of a pathology, mainly based on the cultivation of infectious agents isolated from diseased organisms [106]. These postulates were recently adapted by Fredericks *et al.* [107] and Mokili *et al.* [108] to molecular and NGS data. Metagenomics is a powerful tool to detect potentially new or re-emerging viruses in complex samples, however, subsequent studies are needed to determine if the viruses that were detected represent a potential threat to human (or animal) health. Defining the causality of a given pathology is a complex task, and the isolation of viral agents via cell culture or intracerebral inoculation of suckling mice remains the gold-standard in conducting studies of the pathogenicity of viruses detected by metagenomics.
